# A Relaxed Horse—A Relaxed Client? An Experimental Investigation of the Effects of Therapy Horses’ Stress on Clients’ Stress, Mood, and Anxiety

**DOI:** 10.3390/ani14040604

**Published:** 2024-02-13

**Authors:** Alicia Müller-Klein, Moritz Nicolai Braun, Diana S. Ferreira de Sá, Tanja Michael, Ulrike Link-Dorner, Johanna Lass-Hennemann

**Affiliations:** 1 Division of Clinical Psychology and Psychotherapy, Department of Psychology, Saarland University, 66123 Saarbrücken, Germany; aliciaklein@gmx.net (A.M.-K.); moritz.braun@uni-saarland.de (M.N.B.); diana.ferreira@uni-saarland.de (D.S.F.d.S.); t.michael@mx.uni-saarland.de (T.M.); 2 Institut für Tiergestützte Ausbildung und Therapie (ITAT), Eschringerstraße 70, 66131 Saarbrücken, Germany

**Keywords:** equine, horse, welfare, interspecies communication, therapy, emotional contagion

## Abstract

**Simple Summary:**

Equine-assisted therapies are becoming increasingly popular for addressing physical and psychological disabilities in clients. The role of the horse’s welfare in equine-assisted service receives increasing attention in research. Recent research has shown that horses are able to perceive human emotions and respond to human stress responses. However, no research has yet looked at the other side of the coin—whether and how humans perceive and react to equine stress levels during equine-assisted services. In our study, healthy participants interacted once with a stressed horse and once with a relaxed horse and we asked them before and after the interactions about their mood and their current anxiety level. We also assessed participants’ and horses’ heart rates. The results of our study showed that the stressed horses were stressed during and after the stress manipulation. Participants reported better mood and less anxiety after the interaction with both the previously stressed and the relaxed horse. Thus, our data suggest that humans cannot intuitively recognize the emotional states of horses. Practical implications for equine-assisted services and other equestrian activities are discussed.

**Abstract:**

Equine-assisted therapies are becoming increasingly popular for addressing physical and psychological disabilities in clients. The role of the horse’s welfare in equine-assisted service receives increasing attention in research. Several studies have shown that horses are able to perceive human emotions and respond to human stress responses. However, no research has yet looked at the other side of the coin—whether and how humans perceive and react to equine stress levels during equine-assisted services. To fill this gap in the research, we employed a within-subjects design, in which horse-naïve participants had a standardized interaction with both an experimentally stressed horse and an experimentally relaxed horse. We assessed physiological indicators of stress (heart rate, heart rate variability, and salivary cortisol) in participants and horses, as well as psychological indicators of stress (state anxiety and positive and negative affect) in participants. Although our stress and relaxation manipulations were successful (indicated by horses’ physiological indicators of stress), we did not find any difference in the participants’ physiological or psychological indicators of stress between the interaction with a stressed and the interaction with a relaxed horse. Together with results from previous studies, this suggests that humans cannot intuitively recognize the (physiological) stress level of horses, which has important implications for effective communication and bonding between humans and horses and for the safety of equine activities.

## 1. Introduction

Equine-assisted services (EASs) [1] are becoming increasingly popular for addressing physical and psychological disabilities in clients. Although there is still a lack of rigorous, well-controlled studies, some evidence suggests that EASs have a beneficial effect on clients with physical and psychological disabilities [2].

In the early years of animal-assisted interventions (AAIs), those working in the field focused mainly on the human client involved, but in recent years, the welfare of animals in AAIs has received increasing attention. EASs may pose a significant stressor to horses, as clients have a wide variety of disabilities, which may result in poor balance or in uncontrolled physical or verbal actions, and may give cues that are inaccurate or excessive. Furthermore, the horse is constantly on lead, which may be stressful for horses. There is a range of studies in which stress in therapy horses during EASs has been investigated. Some studies did not find increased stress levels in horses during EAS [3,4,5,6,7,8]. In interpreting these findings, it must be considered that some of the studies that report null findings compared EAS with other equestrian activities (e.g., riding lessons for beginners [5,7,8]). Therefore, from these studies it can only be concluded that EASs are not more stressful than other equestrian activities. Contrasting these findings, other studies found increased stress levels in horses when comparing EASs to baseline stress levels [9] and for EASs with specific patient groups [10,11]. Furthermore, research has shown that the use of bits and auxiliary reins may pose a significant stressor to horses [12,13,14,15]. This finding seems crucial, as a significant proportion of handlers in EASs traditionally work with bits and auxiliary reins. Thus, it is reasonable to conclude from the available evidence that EASs may pose stress on therapy horses, at least in some circumstances. 

Recently, it has been argued that the One Health concept, the concept of combined veterinary and human health [16], is an important framework for AAI [17]. In this vein, there is growing consensus that ethically justifiable AAI should foster health and wellbeing in both humans and animals, while avoiding any risks for and any suffering in both [18,19]. Thus, ethically justifiable EASs should aim to avoid stress in therapy horses. 

Apart from the horse welfare perspective, equine stress levels might also be related to the quality and success of EASs. If clients were sensitive (i.e., perceive and react) to horses’ stress levels, equine stress levels may directly influence the quality and success of the EAS. Research from human–human interactions show that emotions can be contagious [20,21,22]. Emotional contagion can be reflected in showing a similar facial, vocal or postural expression as well as similar neurophysiological and neurological reactions [23]. As a response to emotional contagion, individuals show behavioral, attentional, and emotional synchrony [24]. Anecdotal evidence suggests that emotional contagion also takes place between animals and humans and recent research indeed supports the notion that horses are sensitive to human emotions [25,26,27]. For instance, Keeling and colleagues [28] asked horse handlers to walk a defined route with their horse several times. The handlers were warned that the horses would be startled at a certain point. Although the startle stimulus did not occur, an increase in heart rate was measured in both the handlers and the horses. Although the horses could not have known about the announced startle stimulus, they reacted to their handler’s tension with increased stress levels. Importantly, this line of research does not necessarily imply that the horse understands human emotions or feels the same emotions, it is more likely that the horse is responding to the change in the human’s body posture or movements.

Nevertheless, these studies show that horses are sensitive to the emotional state of their handler/rider, i.e., they perceive it and react to it. But what about the other side of the coin? Are clients sensitive to the therapy horses’ stress level? And if so, does the equine stress level impact the stress and well-being of the client?

So far, the perception of the One Health concept regarding EASs was that EASs are believed to be helpful for clients and that professionals applying EASs should ensure that it does not harm the employed animals. However, if our assumption that equine stress levels have a direct impact on clients’ stress levels during EAS can be verified, this implies that equine wellbeing during EASs is not only important from an ethical point of view, but that it may directly influence the quality and success of EASs. 

There is a lack of research on whether and how humans perceive and react to equine stress levels during EASs. However, some research has focused on the ability to identify equine affective states. Two studies, focused on the interpretation of equine acoustic communicative signals (whinnies). Both studies showed that horse-experienced and horse-inexperienced participants were able to identify the valence and arousal of horses’ vocalization above chance level [29,30]. Other studies focused on the human ability to identify equine affective states in horses’ body language [31,32,33,34]. Taken together, these findings suggest that previous experience with horses and empathy influence the ability to identify affective states in horses. However, identification rates in the above referenced studies were far from perfect. It is important to note that all of these studies used either photographs, videos or audio files of horses’ communicative signaling, i.e., none of these studies investigated a real interaction between a participant and a horse, which might be a prerequisite for emotional contagion to take place.

To summarize, EASs are frequently practiced and, although the evidence is sparse, some results indicate that patients may benefit from EASs. Research in the last ten years increasingly focused on the wellbeing and stress of horses during EASs. However, there is a lack of studies focusing on the effects of the horses’ stress levels on the wellbeing of the client and the quality of the EASs. Considering the emerging consensus in the field, that the One Health concept should be adapted to AAI, these aspects seem crucial to consider. The present study fills this gap. In our study, horse-naïve participants (i.e., participants with no (or little) prior experience with horses, which we defined as less than one year of riding lessons which stopped at least ten years prior) had a standardized interaction with both an experimentally stressed horse and an experimentally relaxed horse. We assessed physiological markers of stress in horses (heart rate, heart rate variability, and salivary cortisol) as well as physiological (heart rate, heart rate variability, and salivary cortisol) and psychological measures (state anxiety and positive and negative affect) in participants. Heart rate (HR) and heart rate variability (HRV) have been extensively employed in human and animal stress research, with a higher HR and a lower HRV indicating a shift of the autonomic nervous system towards a sympathetic dominance and low parasympathetic activity, which is a clear indicator of a stress response [35,36]. Cortisol is a steroid hormone, which is released in response to stress and has been used as a stress marker (with higher cortisol levels indicating a more pronounced stress response) in a variety of studies in animals and humans [37,38]. While an increased HR and a decreased HRV is part of the first wave of stress, the cortisol response constitutes the second wave of stress, which is much slower to increase (reaches its maximum about 20–30 min after stressor onset) and decrease (returns to baseline about 60 min after stressor onset) [39,40,41]. We chose state anxiety and positive and negative affect as psychological measures to assess participants’ affective reaction to the interaction, since both measures are frequently used in experimental studies on AATs and human–animal interaction [42,43,44]. 

We hypothesized a significant difference in participants’ physiological and psychological responses between the interaction with a stressed and the interaction with a relaxed horse. Precisely, for interactions with a stressed horse, we hypothesized that state anxiety, negative affect, and salivary cortisol increase during the interaction, whereas the positive affect decreases and that these effects persist for some time after the interaction. In contrast, for interactions with a relaxed horse, we hypothesized that state anxiety, negative affect, and salivary cortisol decrease during the interaction, whereas the positive affect increases, with these effects persisting for some time after the interaction. Further, we hypothesized that, for interactions with a stressed horse, human HR increases and HRV decreases during the interaction and that these effects return to baseline once the interaction is over. In contrast, for interactions with a relaxed horse, we hypothesized that human HR decreases and HRV increases during the interaction, with these effects returning to baseline once the interaction is over.

## 2. Materials and Methods

All data and code can be found in the Open Science Framework project associated with this study (https://osf.io/jk648/). This study was approved by the veterinary office of Saarland (Landesamt für Verbraucherschutz Saarland) and our university’s ethics committee (reference number 17-12).

### 2.1. Sample Recruitment and Sample Characteristics

Our final sample consisted of 42 female students (*Mean*_Age_ = 22.67 years, *SD*_Age_ = 3.16 years, range: 18 years to 33 years) of Saarland University, Germany. Potential participants responded to advertisements circulated at the university campus and via social media. Psychology students received course credit for their participation. Participants were suitable for inclusion if they met the following criteria: healthy (assessed via a short health questionnaire which asked for acute and chronic physical and psychological disorders), female gender, no fear of horses, no animal hair allergy, body weight under 75 kg (weight limit for the therapy horses), and no (or little) prior experience with horses (defined as less than one year of riding lessons, which stopped at least ten years prior to study participation). We checked for the inclusion criteria during a telephone screening. A priori sample size calculation using G*Power (Version 3.1.9.7) [45,46] yielded a sample size of *n* = 36 participants to detect a medium effect size with a power of 90% at an α-level of 0.05. A total of 44 participants met our inclusion criteria and we decided to collect data from all of them to increase our power. However, we had to exclude 2 participants due to poor weather conditions (i.e., heavy rain and storm which came up on the day of the scheduled session) during testing. Thus, our final sample consisted of 42 participants. All participants gave their written informed consent (on the procedure and the measures that were to be collected) prior to the start of their experimental session. Participants were blind to our concrete hypotheses, but they were informed that they were participating in a study on the effects of interactions with horses on physiological stress levels and subjective well-being. All of the experimental sessions took place between 11 a.m. and 02 p.m. at the grounds of the Stone Hill Ranch, Saarbrücken, Germany. 

### 2.2. Design

Our study consisted of a within-subject design, in which horse-naïve participants had a standardized interaction with both an experimentally stressed and an experimentally relaxed horse. The order of the interactions was counterbalanced across participants. We assessed physiological stress markers (heart rate, heart rate variability, and salivary cortisol) in the horses. Furthermore, we assessed physiological (using heart rate, heart rate variability, and salivary cortisol) and psychological indicators of stress (state anxiety and positive and negative affect) in the participants.

### 2.3. Materials and Measures

#### 2.3.1. Positive and Negative Affect Schedule (PANAS)

The German version of the Positive and Negative Affect Schedule (PANAS) was used to assess positive and negative mood before and after the interaction with each horse [47]. In detail, each participant completed the PANAS five times: before the interaction with the first horse (baseline), immediately after the interaction with the first horse (interaction first horse), and approximately 30 min after the interaction with the first horse (post first horse; this second post measurement also served as the baseline measurement for the second horse contact (baseline 2)), immediately after the interaction with the second horse (interaction second horse), and 30 min after the interaction with the second horse (post second horse). The PANAS questionnaire consists of 10 positive affects (PA: interested, excited, strong, enthusiastic, proud, alert, inspired, determined, attentive, and active) and 10 negative affects (NA: distressed, upset, guilty, scared, hostile, irritable, ashamed, nervous, jittery, and afraid). Participants were asked to rate each item on a scale from 1 to 5, based on the strength of their current emotion, ranging from ‘1 = very slightly or not at all’ to ‘5 = extremely’. For each of the PANAS domains, scores can range between 10 and 50, with higher scores on the positive or negative domain indicating greater PA or NA, respectively. Cronbach’s alpha was 0.88 for the positive domain and 0.81 for the negative domain (mean Cronbach’s alpha across the five timepoints). 

#### 2.3.2. State-Trait Anxiety Inventory (STAI-T and STAI-S)

The German version of the State-Trait Anxiety Inventory (STAI) [48,49] was used to assess trait anxiety (STAI-T) once online before the experimental session as well as state anxiety (STAI-S) before and after the interaction with each horse. In order to determine a change in state anxiety depending on the condition, participants completed the STAI-S at the same five times they completed the PANAS. Both of the STAI scales are brief self-report questionnaires consisting of 20 items each. Participants were asked to rate each item on a 4-point Likert scale. The sum scores of both scales range from 20 to 80, while lower scores are indicators of low (state or trait) anxiety and higher scores indicate high (state or trait) anxiety. Cronbach’s alpha was 0.88 for the STAI-T and 0.84 for the STAI-S (mean Cronbach’s alpha across the five timepoints). We assessed trait anxiety as a potential moderator of the hypothesized difference in participants’ physiological and psychological responses between the interaction with a stressed and the interaction with a relaxed horse.

#### 2.3.3. Beck Depression Inventory II (BDI-II)

The German version of Beck Depression Inventory–II (BDI-II), a 21-item, self-report measure with scores ranging from 0 to 63 [50], was used to assess the severity of depressive symptoms over the last two weeks, with higher total scores indicating more severe depressive symptoms. Cronbach’s alpha was 0.83. We assessed the depressive symptoms as a potential moderator of the hypothesized difference in participants’ physiological and psychological responses between the interaction with a stressed and the interaction with a relaxed horse. Participants completed the BDI–II online before the experimental session. 

#### 2.3.4. Brief Symptom Inventory (BSI)

The German version of the Brief Symptom Inventory [51] was used to assess the current burden on general mental health. The BSI is a 53-item, self-report measure that assesses symptomatic distress using nine subscales. For the purpose of the current study, only the global severity index (GSI) was used to indicate general mental health problems. Cronbach’s alpha was 0.95. We assessed the GSI as a potential moderator of the hypothesized difference in participants’ physiological and psychological responses between the interaction with a stressed and the interaction with a relaxed horse. Participants completed the BSI online before the experimental session.

#### 2.3.5. Pet Attitude Scale (PAS)

The German version of the Pet Attitude Scale (PAS) [52] was used to assess general attitudes towards pets. In the pet attitude scale, 18 items are rated on a seven-point scale (from 1 = not at all to 7 = totally agree). The German translation of the PAS is based on the modified version of the PAS [53], which assesses the attitude towards pets in pet-owners and non-pet owners. In the modified version, pet owner-specific items are supplemented by an “or would if I had one” (e.g., My pet means more to me than any of my friends (or would if I had one)) to make the PAS relevant for pet-owners and non-pet-owners. Participants can reach a maximum score of 126 and a minimum score of 18 points. The internal consistency, retest reliability, and construct validity of the scale are high [53,54,55]. Cronbach’s alpha was 0.86. We assessed the general attitude towards pets as a potential moderator of the hypothesized difference in participants’ physiological and psychological responses between the interaction with a stressed and the interaction with a relaxed horse. Participants completed the PAS online before the experimental session.

#### 2.3.6. Lexington Attachment to Pets Scale (LAPS)

The German version of the Lexington Attachment to Pets Scale [56] was used to assess emotional attachment to one’s pet. The scale was only administered to participants reporting that they currently owned a pet. The scale consists of 23 items, which are rated on a 4-point scale. Higher scores indicated a stronger attachment to one‘s pet. Cronbach’s alpha was 0.86. We assessed the emotional attachment to one’s pet as a potential moderator of the hypothesized difference in participants’ physiological and psychological responses between the interaction with a stressed and the interaction with a relaxed horse. Participants completed the LAPS online before the experimental session.

#### 2.3.7. Interpersonal Reactivity Index (Saarbrücker Persönlichkeitsfragebogen, SPF)

The German version of the Interpersonal Reactivity Index (SPF, Saarbrücker Persönlichkeitsfragebogen [57], original version [58]) was used to assess empathy in participants. The SPF consists of 16 items that are rated on a 5-point Likert scale (from 1 = do not agree at all to 5 = totally agree). All items are summed up to a total score, with higher scores indicating higher empathy levels. Cronbach’s alpha was 0.81. We assessed empathy as a potential moderator of the hypothesized difference in participants’ physiological and psychological responses between the interaction with a stressed and the interaction with a relaxed horse. Participants completed the SPF online before the experimental session.

#### 2.3.8. Physiological Measurements

##### Heart Rate and Heart Rate Variability (Horses)

The polar V800 equine sensor (Polar Electro Oy, Kempele, Finland) was used to assess HR in horses. A Polar equine science belt (Polar Electro Oy, Kempele, Finland) was located as close to the elbow as possible. Electrocardiogram (ECG) conductive gel was liberally applied to the electrodes of the Polar belt to ensure uniform contact. The belt had a Polar H7 sensor fitted. HR and HRV were analyzed and calculated using the Kubios Software (Kubios HRV Standard 3.4.1, Kubios Oy, Kuopio, Finland). The Kubios software allows users to correct artifacts within the data. This was achieved using a threshold-based artifact-correction algorithm, which compared every instantaneous interbeat interval (IBI) value against the local median interval calculated from 30 successive beats. We employed a custom level 0.3 artifact correction, which has previously been used for artifact correction in equine studies [59,60]. This correction identifies IBI intervals that differ from the median by more than 30% as artifacts. After the removal of the identified intervals, the program’s algorithm substitutes detected errors with interpolated intervals calculated from the differences between previous and next accepted IBI intervals. Further HRV analysis in the time domain was conducted with Kubios, and the root mean square of the successive differences in IBIs (RMSSD) was exported for the six minute resting phases and the successive two minute intervals of horse interaction. Due to technical issues, we could not secure the physiological data for six of the experimental sessions.

##### Heart Rate and Heart Rate Variability (Humans)

ECG was acquired with a personalized wireless BioSignalsPlux Explorer portable device and recorded using OpenSignals (PLUX wireless biosignals S.A., Lisboa, Portugal) at a sampling rate of 1000 Hz. The ECG was collected with a single-lead differential bipolar ECG sensor connected to three, 24 mm disposable Ag–AgCl electrodes (Kendall™ H124SG, Covidien Deutschland GmbH, Halberstadt, Germany). Electrode placement was equivalent to a standard medical-grade V6 lead. Additionally, an ergonomic handheld manual button was used to mark the different events during the experiment. R-waves were automatically detected and manually edited for artifacts, false positives or non-recognized R-waves using the Autonomic Nervous System Laboratory (ANSLAB) version 2.6 [61]. The ECG was then transformed into instantaneous IBIs and instantaneous HR. Further HRV analysis in the time domain was conducted with ANSLAB, and the RMSSD was exported for the six minute resting phases and the successive two minute intervals of horse interaction. For time domain analysis, and specifically RMSSD, it has been previously shown that short measurement intervals of two minutes are sufficient to obtain a reliable and accurate measure of HRV [62,63]. Due to technical issues, we could not secure the ECG data of fourteen of our participants. Further, we had to exclude the data of two additional participants due to noise and missing data, resulting in a total of 26 participants for the ECG analyses.

##### Salivary Cortisol (Horses and Humans)

To assess salivary cortisol as an endocrine stress marker, participants provided five saliva samples: (1) after the first baseline assessment of PANAS and STAI-S, (2) after the interaction with the first horse, (3) before interaction with the second horse/30 min after interaction with the first horse, (4) after interaction with the second horse, and (5) 30 min after interaction with the second horse. For each horse, three saliva samples were taken as endocrine stress makers: (1) during the preparation of the horses, (2) after the interaction with the participant, and (3) 30 min after interaction with the participant. Thus, saliva samples of participants and horses were collected at the same time point, with the time point of the third saliva sample of the first horse corresponding to the time point of the first saliva sample of the second horse. Further details on the time points for the collection of saliva can be found in the Section 2.4. 

We used Salivette tubes (Sarstedt AG, Nümbrecht, Germany) made for the assessment of salivary cortisol to collect saliva from the participants and the horses. In detail, participants were asked to put the cotton swab for saliva collection into their mouth and gently chew on it for about a minute and then return it into the Salivette tubes. 

The procedure for saliva cortisol collection of the horses slightly differed. We practiced saliva collection with the horses several times before the study, i.e., horses were habituated to saliva collection. The horses were not given any feed except for hay before the study. Using fresh, disposable gloves, we used a cotton swab (Salivette tubes, Sarstedt AG, Nümbrecht, Germany) to collect the horses’ saliva by hand, in the area of the jaw, the cheek pouches and between the rows of teeth and the upper and lower lip. The cotton swab was moved around the horse’s mouth consistently for 60 to 90 s. The swabs were then returned to the Salivette tubes. Saliva samples of horses and participants were frozen at −20 °C until analysis in the laboratory. After thawing the saliva samples for biochemical analysis, the fraction of free cortisol in saliva was determined using a time-resolved immunoassay with fluorometric detection as described in detail elsewhere [64].

#### 2.3.9. Horses

##### Selection of Horses and Matching of Horses

**Figure 1:**
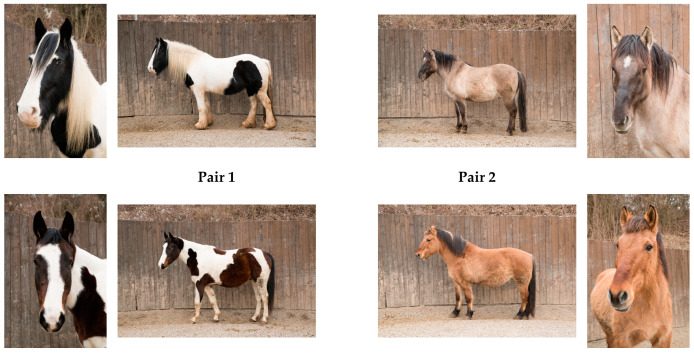
The four horses. Participants either interacted with two piebald or with the two buckskin horses in a standardized 6-min interaction (2 min of petting/scratching the horse, 2 min of sitting on the horse while the horse was standing still, and 2 min of riding the horse in walk while the experimenter was leading the horse). Before the interaction, one of the horses was experimentally stressed, while the other was relaxed. Please see the main text for further details.

Three healthy geldings and one mare of various breeds (Polish Warmblood, Quarter Cross, Tinker, Konik horse) aged 8 to 16 took part in the experiment. All of the horses were well trained and experienced therapy horses (with three to five years of experience as therapy horses). The four horses that took part in the study were usually worked bitless and without any auxiliary reins on four to five days a week with one to two workouts per day (with breaks) of both riding (in the arena/round pen, as well as cross country rides) and groundwork (with varying demands). The horses had species-appropriate living conditions in a mixed herd with free access to hay and were returned to their herd directly after the experimental sessions. Each horse took part in no more than one experimental session per day and did not participate in EAS sessions or other equestrian activities on the days of the experimental session. Horses were matched in two pairs according to their color (two piebald horses, two buckskin horses) and size (see Figure 1). Thus, each participant interacted with two very similar looking horses, in order to control for possible preferences for colors or types of horses. The allocation of horses to the stressed and relaxed conditions was also balanced, resulting in a total of eight different possible combinations between the pairs of horses, the order of the conditions (stressed vs. relaxed horse), and the allocation of the horses within a pair to the conditions. 

### 2.4. Procedure

Overall, the study consisted of three different parts (1) telephone screening, (2) online questionnaire, and (3) the experimental session at ranch grounds.

#### 2.4.1. Telephone Screening

During the telephone screening, participants were briefly informed about the study and the inclusion criteria were checked. Participants who met the inclusion criteria and were willing to participate in the study were asked to complete an online questionnaire. Further, an appointment was made for the experimental session.

#### 2.4.2. Online Questionnaire

The online questionnaire consisted of a background questionnaire (age, pet ownership) and we assessed trait anxiety (STAI-T), depressive symptoms (BDI–II), general psychopathological symptoms (BSI), general attitudes towards pets (PAS), attachment to pets (LAPS; only for pet owners), and empathy (SPF) as potential moderators of the hypothesized difference in participants’ physiological and psychological responses between the interaction with a stressed and the interaction with a relaxed horse. 

#### 2.4.3. Experimental Session

##### Experimental Location

The experimental sessions took place at the Stone Hill Ranch, the cooperation stable of the ISAAT-certified Institut für Tiergestützte Ausbildung und Therapie (institute for animal assisted training and therapy), Saarbrücken Germany. Approximately 20 horses are stabled there of which 10 are regularly employed in EASs. 

##### Arrival at the Ranch and Baseline Measurement

**Figure 2:**
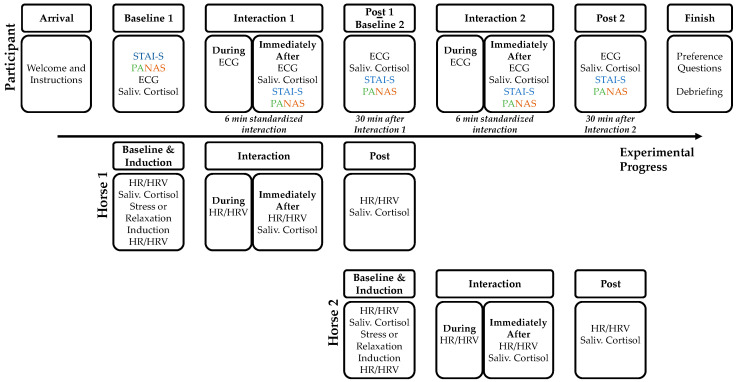
The course of an experimental session for the human participant and the two horses is illustrated. See the main text for details.

Participants were asked to refrain from physical exercise, caffeine, alcohol, and nicotine consumption on the day of the experimental session (as these activities may influence the participants’ cortisol levels [65,66,67]). Each participant was tested individually with no other participants present during the experimental session. On the day of the experimental session, participating horses were not worked before the experimental session. Upon arrival at the ranch, participants were greeted by the experimenter and led into a room from which the horses could not be seen. Participants were then prepared for the measurement of the ECG and asked to fill out the questionnaires on mood and state anxiety (PANAS, STAI-S) for the baseline assessment. After completing the questionnaires, a six minute baseline ECG was conducted, during which participants were asked to be in a comfortable sitting position and to breathe slowly and evenly. Furthermore, participants provided the first saliva sample for the assessment of salivary cortisol. The course of the experimental session for participants and the two horses is depicted in Figure 2.

##### Standardized Interaction with the First Horse

After completing the baseline measurement, participants were led to an enclosed round pen with a three-meter-high wooden fence, which completely obscured the horses‘ and participants’ view of the surroundings, to avoid disturbance during the experimental sessions. In this round pen, the stress/relaxation induction had already taken place for the first horse (see the section *Preparation of the horses* below for details) for the interaction with the participants. The horse handler was waiting with the (previously stressed or relaxed) horse in the round pen. The experimenter stayed with the horse and the participant, while the horse handler left the round pen to prepare the second horse. The horse was equipped with a rope halter, a lead, and a bareback pad (see the section *Preparation of the horses* below). Participants were then introduced to the horse, i.e., participants were told the name of the horse and if questions regarding the horse (e.g., on horses sex or race) arose, the experimenter briefly answered these. Afterwards, the standardized six minute interaction with the horse began. This standardized interaction consisted of two minutes of petting/scratching the horse, two minutes of sitting on the horse while the horse was standing still, and two minutes of riding the horse in walk while the experimenter was leading the horse. During the two minutes of petting/scrachting, participants were asked to stand at the side of the horse (approximately shoulder height) and were told that they were allowed to stroke and cuddle (the neck and shoulder area, but not the head, especially the area around the mouth and nostrils). The experimenter stood near the horse’s head with a loose rope in his hand so that the horse could move its head and interact with the participant, but the experimenter could intervene if a dangerous situation arose. After the two minutes of petting, participants were asked to mount the horse with a three-step mounting aid. The participants were asked to concentrate on the horse during the intervention and not to talk to the experimenter (talking to the horse was allowed) unless there was a reason (e.g., discomfort, desire to stop). The HR of the horse as well as the ECG of the participant were continuously measured during this standardized six minute interaction. 

##### First Waiting Period

After the completion of the standardized interaction with the first horse, participants returned to the room in which the baseline assessment had taken place. After arrival at the room, an ECG was conducted again for six minutes. Further, participants were asked to fill out the PANAS and the STAI-S again and to provide a second saliva sample. During the waiting period, participants were allowed to read magazines and to drink water. Magazines were provided by us and were chosen to be neutral in content (magazines on lifestyle, cooking, nature). Furthermore, participants were not allowed to use their phones during the waiting period. The restriction of telephone use and the content of the magazines was chosen to reduce the chance of changes in mood/affect that were not related to the experimental manipulation. Thirty minutes after the interaction with the first horse, a third six minute ECG was conducted, participants provided the third saliva sample, and filled out the STAI-S and the PANAS again. At the same time, HR was assessed, and a saliva sample was taken from the first and the second horse. Afterwards, participants were led to the round pen again.

##### Standardized Interaction with the Second Horse

The standardized interaction with the second horse completely paralleled the standardized interaction with the first horse. Participants were again asked to pet the horse for two minutes, sit on the horse for two minutes, and ride on the horse for two minutes while the experimenter was leading the horse. Again, the HR of the horse and the ECG of the participant were continuously measured during this standardized six minute interaction. The only difference between the two conditions was that participants interacted once with a previously stressed and once with a previously relaxed horse, with the order counterbalanced across participants.

##### Second Waiting Period

After the standardized interaction with the second horse, participants were again led to the room. There, the ECG was conducted again for six minutes. Participants again completed the STAI-S and the PANAS and provided their fourth saliva sample. During the waiting period, participants were again allowed to read magazines and to drink water. Thirty minutes after the interaction with the second horse, participants provided their last saliva sample and filled out the STAI-S and the PANAS one more time. Additionally, a final six minute assessment of participants’ ECG was conducted. At the same time, HR was assessed, and a saliva sample was taken from the second horse.

##### Questionnaire on Preference for the Horses and Debriefing

At the end of the experiment, participants answered a self-developed two-item questionnaire on the wellbeing of the horses and on the participants’ preferences for the horses: Question 1: “Which of the two horses do you think felt better—The first one or the second one?” was answered on a continuous scale from 0 (“the first horse”) to 5 (“the second horse) or anything in between (in case participants had a tendency but were not completely decided or did not think that the one or the other felt better). Question 2: “Which of the two horses would you rather interact with again?”. The first question was asked to determine if participants were aware of the horses’ stress level. The second question was asked to test for possible preferences for interacting with relaxed horses. Afterwards, participants were debriefed about the detailed hypotheses of the study, had the opportunity to ask further questions, and were thanked for their participation. 

#### 2.4.4. Experimental Manipulations: Stress vs. Relaxation Induction

The experimental stress and relaxation inductions were conducted in the same round pen in which the standardized interactions between the participants and the horses took place. All participating horses were regularly trained in this round pen and were familiar with the surroundings. 

##### Preparation of the Horses

Prior to the stress/relaxation induction, the horses were prepared for the experimental sessions at the usual grooming site of the Stone Hill Ranch. There, the horse was fitted with the Polar equine science belt to measure their HR during the intervention. A bareback pad was then placed over the equine sensor to fix the sensor in place and to allow the participants to ride during the intervention. The horses were then brought into the round pen with a rope halter and a lead.

##### Baseline Measurement

When the horse and the horse handler arrived at the round pen, the horse handler took the first saliva sample. Afterwards, the baseline measurement of the horse’s HR started. HR was assessed for six minutes. During this measurement, the horse was standing for the first four minutes and walking (led by the horse handler) for the last two minutes. After the baseline measurement, the horse was either prepared for the stress condition or for the relaxation condition.

##### Stress Induction

To stress the horses, each horse was lunged with a simple snaffle with a single jointed mouthpiece and side reins (head on vertical position) for ten minutes. Horses were lunged on a lunge line and pressure was maintained with a lunging whip. Lunging and the use of bits has been shown to be a significant stressor for horses [12,13,14,15,68]. We chose this type of stressor because lunging and the use of bits and auxiliary reins is a realistic stressor for horses employed in EASs. Furthermore, the length and intensity can be controlled and the horses used in our study were usually worked bitless and without auxiliary reins (assuring that the stressor was comparably new to all four horses). After the baseline measurement, the bridle and side reins were attached in the round pen. The horse was then lunged for ten minutes (five minutes active trot, five minutes canter). The horses were constantly pressured to maintain their speed. The horses’ HR was continuously assessed during the stress induction procedure.

##### Relaxation Induction

To relax the horses after the baseline measurement, the rope halter was taken off and the horse was allowed to move freely in the round pen and to explore it for ten minutes. When the horse decided to approach the horse handler during the ten minutes relaxation period, the horse handler petted and massaged the horse according to the particular horse’s preferences. Getting a break and freely moving around [69,70] as well as scratching and petting [71,72] has been shown to be relaxing to horses. As the horses partly excreted sweat in the stress condition, relaxed horses were moistened with a wet cloth under the mane and along the chest before contact with the participant in order to exclude differences in the coat when stroking the animals.

### 2.5. Data Analyses

To check whether our stress and relaxation manipulation was successful, we (1) compared horses’ HR and RMSSD at baseline with horses’ HR and RMSSD during manipulation and (2) compared the stressed and relaxed horses’ HR and RMSSD during baseline, manipulation, and interaction. As Shapiro–Wilk tests and Q–Q plots indicated that the horses’ HR and RMSSD were not normally distributed, we computed Wilcoxon signed-rank tests for comparisons (1) and Mann–Whitney U tests for comparisons (2). Due to poor data quality, we decided to refrain from analyzing horses’ cortisol level. In detail, several samples showed unrealistically high cortisol levels (>150 nmol/L, which are not likely to be within the normal physiological range [73,74]), unrealistically low cortisol levels (zero or close to zero nmol/L) or unrealistic changes from one point of measurement to the next (~100 nmol/L change or more within 30 min).

To analyze changes in state anxiety, positive affect, negative affect, and salivary cortisol due to the interactions with the horses, we used the multivariate approach to repeated measures analysis with a-priori-specified contrasts according to our hypotheses, thereby transforming the tripartite factor Time (Baseline vs. Interaction vs. Post Interaction) into a vector of two orthogonal contrast variables [75]. That is, the first contrast compared the scores immediately after the interaction with the horse with those 30 min after the interaction with the horse. The second contrast compared the averages for scores immediately after the interaction with the horse and scores 30 min after the interaction with the horse with those at baseline. We computed this analysis including the factor Horse (Stressed vs. Relaxed), which, in case of a significant interaction for Time × Horse, we followed up by two separate analyses, one for the relaxed and one for the stressed horse and by moderator analyses including the potential moderators (see Section 2.3).

To analyze changes in human HR and RMSSD, due to the interactions with the horses, we again used the multivariate approach to repeated measures analysis with a-priori-specified contrasts, according to our hypotheses. This time, we employed polynomial contrasts to transform the sexpartite factor Time (Baseline vs. InteractionFirstPart vs. InteractionSecondPart vs. InteractionThirdPart vs. Post1 vs. Post2) into a vector of five orthogonal contrast variables. We again computed this analysis including the factor Horse (Stressed vs. Relaxed), which, in case of a significant interaction for Time × Horse, we followed up by two separate analyses, one for the relaxed and one for the stressed horse. To account for any differences in nerval reactivity between the participants, we *z*-standardized the HR and RMSSD scores for each participant before computing the described analyses. However, the pattern of results was the same for *z*-standardized and non-standardized data (see the R script in the OSF project).

For all statistical analyses, we applied the significance criterion of *p* < 0.05. We analyzed the data using R (Version 4.0.4) [76] and R Studio (Version 1.4.1106) [77]. Please see the analysis script for the specific packages we used.

## 3. Results

### 3.1. Manipulation Check

**Figure 3:**
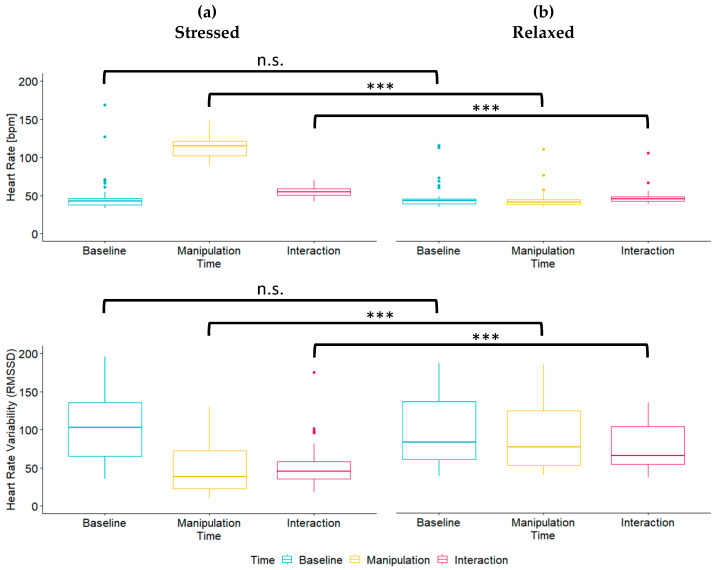
Boxplots of horses’ HR and RMSSD for (**a**) the stress and (**b**) the relaxation manipulation. Significances of the differences in HR and RMSSD between the relaxed and the stressed horses are depicted. Note that not all of them appear senseful after the data have been collected. *** = *p* < 0.001. n.s. = not significant.

The Wilcoxon signed-rank tests indicated that the stress manipulation led to a significant increase in horses’ HR (*W* = 4, *p* < 0.001) and to a significant decrease in horses’ RMSSD (*W* = 616, *p* < 0.001; Figure 3a). By contrast, the relaxation manipulation did not have any significant effects on horses’ HR (*W* = 410, *p* = 0.123) or RMSSD (*W* = 377, *p* = 0.318; Figure 3b). Importantly, while there were no significant differences between relaxed and stressed horses for HR (*W* = 669, *p* = 0.819) or RMSSD (*W* = 589, *p* = 0.512) at baseline, stressed horses had a significantly higher HR (*W* = 13, *p* < 0.001) and a significantly lower RMSSD (*W* = 988, *p* < 0.001) than relaxed horses during the manipulation, which persisted during the interaction (HR: *W* = 254, *p* < 0.001; RMSSD: *W* = 948, *p* < 0.001). This suggests that our stress and relaxation manipulations were successful.

### 3.2. State Anxiety, Negative and Positive Affect, and Human Salivary Cortisol

**Figure 4:**
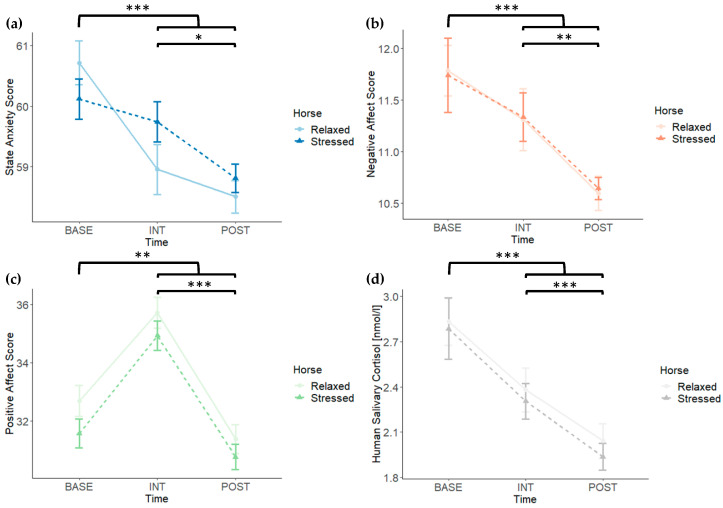
Data for (**a**) state anxiety (STAI-S), (**b**) negative affect (PANAS), (**c**) positive affect (PANAS), and (**d**) human salivary cortisol are depicted at baseline (BASE), immediately after the respective interaction (INT), and 30 min after the respective interaction (POST). Error bars depict standard errors. For all four measures, only the main effect for Time reached significance (see Table 1). For the sake of completeness, significance of the a priori-defined Helmert contrasts are depicted as well. Note that not all of them appear senseful after the data have been collected. * = *p* < 0.05. ** = *p* < 0.01. *** = *p* < 0.001.

For state anxiety, positive and negative affect, and human salivary cortisol, all of our analyses yielded significant main effects for Time (see Table 1), driven by decreases in state anxiety, negative affect, and human salivary cortisol from baseline (*Mean*_STAI-S_ = 60.42, *SD*_STAI-S_ = 2.52; *Mean*_PANAS-NA_ = 11.76, *SD*_PANAS-NA_ = 2.21; *Mean*_SalivaryCortisol_ = 2.81 nmol/L, *SD*_SalivaryCortisol_ = 1.31 nmol/L) to interaction (*Mean*_STAI-S_ = 59.35, *SD*_STAI-S_ = 2.74; *Mean*_PANAS-NA_ = 11.32, *SD*_PANAS-NA_ = 1.94; *Mean*_SalivaryCortisol_ = 2.34 nmol/L, *SD*_SalivaryCortisol_ = 0.96 nmol/L) to post interaction (*Mean*_STAI-S_ = 58.65, *SD*_STAI-S_ = 1.84; *Mean*_PANAS-NA_ = 10.62, *SD*_PANAS-NA_ = 1.01; *Mean*_SalivaryCortisol_ = 1.99 nmol/L, *SD*_SalivaryCortisol_ = 0.75 nmol/L) and an increase in positive affect from baseline (*Mean*_PANAS-PA_ = 32.13, *SD*_PANAS-PA_ = 3.78) to interaction (*Mean*_PANAS-PA_ = 35.32, *SD*_PANAS-PA_ = 3.76) but a decrease in positive affect from interaction to post interaction (*Mean*_PANAS-PA_ = 31.07, *SD*_PANAS-PA_ = 3.35), regardless of the condition (see Figure 4). However, none of our analyses yielded significant main effects for Horse (all *F*s < 3.65, all *p*-values > 0.063; see Table 1) or significant interactions for Time × Horse (all *F*s < 1.86, all *p*-values > 0.169; see Table 1). Given that the interaction for Time × Horse did not reach significance for any of the measures, we refrained from computing the separate analyses for the stressed and the relaxed condition with the orthogonal a priori contrasts and the moderator analyses we initially planned. Instead, we computed a data-driven post hoc contrast to test whether the reduction in state anxiety from baseline to immediately after the interaction was larger in the relaxed than in the stressed horse condition. However, this contrast did not reach significance (*F*(1, 41) = 3.80, *p* = 0.058).

### 3.3. Human ECG Data

**Figure 5:**
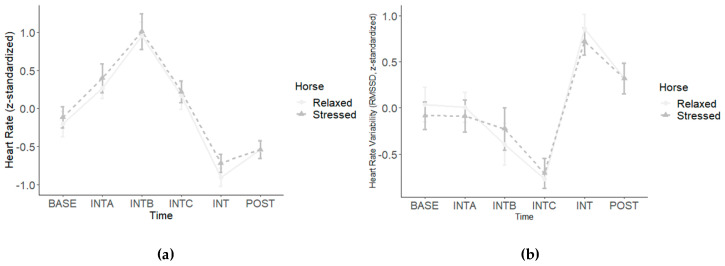
Z-standardized data for human (**a**) heart rate and (**b**) heart rate variability are depicted at baseline (BASE), during the interaction (INTA = 2 min of petting/scratching the horse, INTB = 2 min of sitting on the horse while the horse was standing still, and INTC = 2 min of riding the horse in walk while the experimenter was leading the horse), immediately after the interaction (INT), and 30 min after the interaction (POST). Error bars depict standard errors. Only the main effects for Time reached significance.

For human HR, our analysis yielded a significant main effect for Time (*F*(5, 21) = 22.04, *p* < 0.001), but neither a significant main effect for Horse (*F*(1, 25) = 0.38, *p* = 0.544) nor a significant interaction for Time × Horse (*F*(5, 21) = 0.30, *p* = 0.908). For human RMSSD, our analysis yielded the same pattern of results. Again, there was a significant main effect for Time (*F*(5, 21) = 14.01, *p* < 0.001), but neither a significant main effect for Horse (*F*(1, 25) = 0.04, *p* = 0.852) nor a significant interaction for Time × Horse (*F*(5, 21) = 0.27, *p* = 0.925). Given that the interactions for Time × Horse did not reach significance, we refrained from computing the separate analyses for the stressed and the relaxed condition with the orthogonal a priori contrasts and the moderator analyses we initially planned. The human ECG data are presented in Figure 5.

### 3.4. Questionnaire on Preference for the Horses

Regarding the question “Which of the two horses do you think felt better—The first one or the second one?”, out of our 42 participants, 26 (61.9%) reported that the relaxed horse felt better, whereas 15 (35.7%) reported that the stressed horse felt better. Only one participant was undecided (i.e., reported exactly 2.5 on the scale). A Wilcoxon signed-rank test indicated that on average, participants did not perceive that the relaxed horse felt better than the stressed horse (*W* = 343, *p* = 0.130). Regarding the question “Which of the two horses would you rather interact with again?”, 24 (57.1%) of the participants preferred to interact with the relaxed horse again. However, a binomial test yielded that this preference for the relaxed horse was not significant (*p* = 0.441).

## 4. Discussion

The present study aimed to assess whether humans react to equine stress levels with increased physiological and subjective stress levels. Our results showed that our (physiological) stress induction was successful, i.e., horses in the stress induction condition showed higher HR and decreased HRV during the stress induction and subsequent interaction with the participant compared to relaxed horses. However, contrary to our hypotheses, we did not find any difference in the participants’ physiological or psychological indicators of stress between the interaction with a (physiologically) stressed horse and the interaction with a relaxed horse. Thus, our results show that, at least during a brief interaction, participants do not respond to horses’ stress levels with increased physiological arousal or subjective ratings of increased anxiety or negative affect. Instead, participants’ subjective ratings of anxiety and positive and negative affect indicated that they perceived both interactions with the stressed horse and with the relaxed horse as positive. Furthermore, when asked which horse felt better during the interaction, participants were unable to correctly identify the stressed horse. Moreover, participants did not express a preference for the relaxed horse, when asked which of the two horses they would prefer to interact with again. Thus, our data show that, at least during a brief interaction with a stressed horse, participants do not perceive and react to the stress level of the horse. Instead, participants reported more positive affect, less negative affect, and less state anxiety after interacting with both horses.

However, our results need to be interpreted with caution as we only assessed physiological indicators of stress in horses and did not assess any behavioral markers of stress. Lunging and the use of bits and auxiliary reins (restricting horses’ free movements) have been shown to be an emotional stressor for horses before, with horses showing clear behavioral indicators of stress [12,13,14,15,68]. Thus, we can strictly only say that (while we aimed to induce emotional stress in our therapy horses) we were able to show physiological arousal in our therapy horses.

Some studies on emotional contagion between species have also focused on physiological changes (HR and HRV) only and were able to show that dogs and horses react to human stress levels with a synchronization in HR [28,78]. However, our study focused on the other side of the coin, i.e., the human reaction to physiological changes in horses, and we did not find evidence that participants perceived and reacted to horses’ (physiological) stress level. Our findings are consistent with other studies showing that humans perform far from perfectly in identifying affective states in horses. For instance, two studies on the identification of the valence and arousal of horses acoustic communicative signaling found that participants were able to correctly identify valence and arousal of horses’ whinnies in at most around 64% of the cases [29,30]. Further, in a study by our research group, we found that participants with horse experience correctly identified only about 50% of affective states in horses’ body language and for horse-inexperienced individuals the identification rate was even lower (30%) [34]. Thus, our current study extends the findings from previous studies, in which participants were presented with photographs, videos, or acoustic cues of horses [31,32,33,34] to a real-life horse-participant interaction.

Another possible explanation for our failure to find an effect of the horse’s stress level on participant stress, anxiety, and mood is that each of the participant–horse interactions lasted only six minutes. Because of our complex within-subject design, we chose to assess a short interaction between participants and horses to assure a reasonable length of the whole experimental procedure. However, longer horse–participant interactions might enhance the participant’s “feeling for the horse”, potentially supporting emotional contagion. Further, our data may not be generalizable to real-life EASs, as clients typically and ideally work with one therapy horse over several sessions to build a relationship with it, which again might support emotional contagion. As described above, horse experience and emotion recognition ability/empathy are factors that influence the human ability to identify affective states in horses [29,31,32,33,34]. Ideally, horse experience and empathy are promoted during EASs. Thus, it would be very interesting to investigate differences in the ability to recognize horses’ affective states (and how this may relate to empathy for other humans) before the beginning of participating in EASs and after a substantial time of participating in EASs. Ideally, such a study should also comprise the effects of EASs on the recognition of affective states in horses on different age groups, which seems to be of special relevance as the majority of participants in EAS are children and adolescents, whose empathy skills are still developing [79].

From an animal welfare and health perspective, however, it is important to note that our participants experienced both interactions with the stressed horse and with the relaxed horse as stress- and anxiety-reducing. These findings are consistent with a large body of research that has found a stress-reducing effect from animal contact [80,81]. Our hypotheses were that there is an emotional contagion from horse to human and that interactions with stressed therapy horses have no positive effect on human wellbeing. However, our data suggest that this is not the case. Thus, our findings highlight the importance of training riding therapists and instructors to perceive and interpret the expressive behavior of horses, in order to communicate this to their clients and to secure their horses’ wellbeing and health at all times. This is of particular importance, given that previous research showed that horse trainers working with EAS were not good at predicting the behavior of their horse in several stress tests [82], which indicates that even experienced trainers might at least partially misinterpret the behavior of their horses.

Besides the lack of a behavioral stress assessment, some other limitations of our research have to be taken into account. First, our sample consisted of healthy, young, and horse-inexperienced women. We decided to assess a women-only sample for several reasons. First, the weight limit for our therapy horses is 75 kg. Even though not impossible, it would have been difficult to find many men weighing under 75 kg, who were willing to participate in our study. Second, the majority of students enrolling in psychology classes are women. Thus, we decided to assess women only, because, in our view, a gender-balanced sample would not have been realistic to achieve, and we decided to go for a women-only sample instead of an imbalanced sample. Nevertheless, the generalizability of our findings to other genders and age groups as well as to clients receiving EASs is limited. Second, due to technical issues, we had a substantial loss of data for the human ECG. Even though data loss due to technical problems is common in ambulatory physiology assessments, this limits the interpretability of our human HR and HRV data.

## 5. Conclusions

In our study, emotional contagion from the horse to the human participant did not take place. Instead, our data showed that participants perceived both the interaction with an experimentally stressed horse and the interaction with an experimentally relaxed horse as positive. This suggests that humans cannot intuitively recognize the emotional states of horses, a finding in line with the existing literature. Thus, if we want to employ horses in animal-assisted interventions, it is our duty to learn to be sensitive to the expressive behavior of horses, in order to enable animal-friendly work with horses. This is of utmost importance in light of the One Health concept and the growing consensus that ethically justifiable AAI should foster health and wellbeing in both humans and animals.

## Figures and Tables

**Table 1:** Inferential statistics for state anxiety, negative and positive affect, and human salivary cortisol.

	Time		Horse		Time *×* Horse	
	*F*(2, 40)	*p*	*F*(1, 41)	*p*	*F*(2, 40)	*p*
State Anxiety	34.34	<0.001	0.21	0.648	1.86	0.169
Negative Affect	19.34	<0.001	0.00	0.978	0.02	0.976
Positive Affect	33.30	<0.001	3.65	0.063	0.08	0.919
Salivary Cortisol	17.69	<0.001	0.17	0.686	0.03	0.967

## Data Availability

All data and code can be found in the Open Science Framework project associated with this study (https://osf.io/jk648/).
